# N95 respirator reuse during the COVID-19 pandemic: Healthcare worker perceptions and attitudes

**DOI:** 10.1017/ice.2020.1348

**Published:** 2020-11-26

**Authors:** Valeria Fabre, Sara E. Cosgrove, Francis Catalfumo, Polly Trexler, Melanie Curless, Lisa L. Maragakis, Clare Rock

**Affiliations:** 1 Department of Medicine, Division of Infectious Diseases, Johns Hopkins University School of Medicine, Baltimore, Maryland; 2 Department of Hospital Epidemiology and Infection Control, The Johns Hopkins Hospital, Baltimore, Maryland

*To the Editor—*The global coronavirus disease 2019 (COVID-19) pandemic has led to an unprecedented scarcity of N95 respirators.^1-4^ The Johns Hopkins Hospital (JHH), a 1,162-bed tertiary-care academic hospital in Baltimore, Maryland, cared for >1,000 inpatients with COVID-19 since March 2020. Early in the pandemic, to preserve existing N95 respirator supplies, JHH implemented a contingency capacity strategy of N95 reuse, where an N95 with the protection of a face shield is donned for a single patient contact, then doffed and stored before being used by the same healthcare worker (HCW) for other patient contacts. HCWs were trained in the correct N95 donning and doffing practices, including direct observations and feedback. HCWs were encouraged by hospital leadership to reuse their N95 due to critical shortages; however, they could obtain a new N95 from their department at any time if there was concern for functional or structural integrity (ie, soiling or damage to any part of the N95, or failure to attain an adequate seal on leak testing) of the respirator. This change in practice was unprecedented, so we surveyed HCWs to understand their perceptions, attitudes, and practices regarding the reuse of N95s.

We developed a 16-item questionnaire using a web-based survey tool (Qualtrics, Provo, UT), and we pilot tested it with 7 HCWs to ensure that the questions were understood as intended. In July 2020, the survey was shared with HCWs working on units caring for patients with COVID-19, and among those who had reused an N95 at least once since March 2020. Survey responses were anonymized, each HCW could only respond once, and the survey closed 4 weeks after becoming available. We did not incentivize participation. Responses on a 5-point Likert scale were collapsed into 2 categories (eg, agree/strongly agree and neutral/disagree/strongly disagree)^5^ and were analyzed using nonparametric tests (ie, Fisher’s exact and Wilcoxon-rank sum tests) using STATA version 16.0 software (StataCorp, College Station, TX). A 2-sided *P* value < .05 was considered statistically significant. The study was approved by the Johns Hopkins University Institutional Review Board.

The survey was completed by 294 HCWs (294 of 890, a 33% response rate); 78% were female; and respondents had a median age of 35 years (range, 22–70). Respondents’ roles included nurse (40%), advanced practitioner (28%), attending/resident physician (23%), and other (9%). Clinical department affiliations included medicine (40%), emergency medicine (29%), surgery (18%), anesthesia (6%), and other (7%). Overall, 53% of participants indicated that they felt comfortable with N95 reuse, and 46% used the same N95 for >14 days. However, 75% expressed discontent with N95 reuse as a PPE-conservation strategy (Table 1). Physician, compared with nonphysicians, were more likely to indicate support of N95 reuse (35% vs 21%; *P* < .05); however, they appeared to have reused their N95s less than HCWs in other roles. Also, 66% of respondents stated that they used their N95 3–5 times per work day, and 10% indicated >5 uses per day. Nurses were less likely than other HCWs to report wearing their N95 for shorter periods after donning: 31% of nurses wore the N95 <1 hour per donning compared to 69% of nonnurses (*P* = .01).

For those who replaced their N95s (n = 153 of 224, 68%), commonly cited reasons were mask soilage (24%), head strap breakages (15%), loss of seal (8.5%), or >1 reason (23%).

Physicians were less likely to consistently perform a user safety seal check at time of reuse compared to other roles (44% vs 62%; *P* = .04). Although a seal check is recommended at every N95 donning to identify air leakage from a gap between the wearer’s face and N95, 29% respondents never performed a seal check or were unaware of what a seal check is.

At 5 months into the COVID-19 pandemic, HCWs caring for hospitalized patients were discontent with the need for continued N95 conservation measures. Despite such reservations, most have continued to comply with N95 reuse recommendation. Although 57% of respondents reused the N95 multiple times over a prolonged period, reuse varied by role, with physicians performing fewer donnings and wearing the N95 for fewer hours than nonphysicians.

The finding that 29% of respondents were not aware of or did not consistently perform a user seal check before donning indicates an area for improvement.

Study limitations include selection bias because HCWs who were not enthusiastic about N95 reuse strategy may have felt more motivated to respond. Our study has limited generalizability because the survey was administered at a single academic hospital.

Given the extensive N95 reuse and discontent with the practice reported by HCWs in the setting of N95 shortages, studies are urgently needed to evaluate important safety issues related to N95 conservation strategies, such as the safe number of N95 reuses before failure.

**Table 1.** Survey Answers by Role

Question	Role					*P* Value
	All (N=294)	Physician (N=68)	Advanced Practitioner (N=83)	Nurse (N=119)	Other (N=24)	
Content with the N95 reuse conservation strategy, no. (%)	72 (25)	24 (33)	22 (31)	23 (32)	3 (4)	.04
Comfortable reusing an N95, no. (%)	84 (47)	23 (27)	22 (26)	36 (43)	3 (4)	.07
Frequency of user seal check, no. (%) Always/almost always Sometimes/Half the time Never/Don’t know what a seal check is	154 (57) 39 (14) 77 (29)	13 (33) 28 (18) 23 (30)	10 (26) 45 (29) 17 (22)	12 (31) 68 (44) 2 (42)	4 (10) 13 (8) 5 (6)	.27
N95 donnings/shift on an average clinical workday, no. (%) 1–2 3–5 >5	294 71 (24) 193 (66) 30 (10)	15 (21) 50 (26) 3 (10)	26 (37) 44 (23) 13 (43)	26 (37) 82 (43) 11 (37)	4 (6) 17 (9) 3 (10)	.10
Longest hours N95 worn on an average clinical workday, no. (%) ≤1 h >1–5 h >5 h	107 (36) 109 (37) 78 (27)	29 (27) 26 (24) 13 (17)	42 (39) 24 (22) 17 (22)	33 (31) 46 (42) 40 (51)	3 (3) 13 (12) 8 (10)	.02
N95 replacements needed in 1 week at the peak of COVID-19, no. (%) Never 1–2 times 3–4 times >5 Does not recall	71 (27) 94 (36) 32 (12) 27 (10) 34 (13)	26 (36) 19 (20) 1 (3) 6 (22) 5 (15)	23 (32) 26 (28) 7 (22) 5 (18) 5 (44)	21 (29) 42 (45) 17 (53) 12 (44) 16 (47)	1 (1) 7 (7) 7 (22) 4 (15) 5 (15)	<.01
Longest number of working days that HCW reused same N95, no. (%) 1–3 d 4–7 d 8–14 d >14 d	30 (12) 59 (23) 51 (20) 117 (45)	0 6 (10) 14 (27) 37 (32)	4 (13) 20 (34) 10 (20) 35 (30)	20 (67) 26 (44) 21 (41) 41 (35)	6 (20) 7 (12) 6 (12) 4 (3)	<.01

## References

[1] Ranney ML , Griffeth V , Jha AK. Critical supply shortages—the need for ventilators and personal protective equipment during the COVID-19 pandemic. N Engl J Med 2020;382(18):e41. 32212516 10.1056/NEJMp2006141

[2] Nogee D , Tomassoni AJ. COVID-19 and the N95 respirator shortage: closing the gap. Infect Control Hosp Epidemiol 2020;41(8):958. 32279694 10.1017/ice.2020.124 PMC7205548

[3] Rebmann T , Vassallo A , Holdsworth JE. Availability of personal protective equipment and infection prevention supplies during the first month of the COVID-19 pandemic: a national study by the APIC COVID-19 task force. Am J Infect Control 2020. doi: 10.1016/j.ajic.2020.08.029. PMC7448742 32858092

[4] Patel AB , O’Donnell A , Bonebrake A , et al. Stewardship of personal protective equipment (PPE): an important pandemic resource for PPE preservation and education. Infect Control Hosp Epidemiol 2020. doi: 10.1017/ice.2020.311. PMC7417984 32576296

[5] Jeong HJ LW . The level of collapse we are allowed: comparison of different response scales in safety attitudes questionnaire. Biom Biostat Int J 2016;4:128–134.

